# Artificial Intelligence Technologies for COVID-19 De Novo Drug Design

**DOI:** 10.3390/ijms23063261

**Published:** 2022-03-17

**Authors:** Giuseppe Floresta, Chiara Zagni, Davide Gentile, Vincenzo Patamia, Antonio Rescifina

**Affiliations:** Dipartimento di Scienze del Farmaco e della Salute, Università di Catania, Viale A. Doria 6, 95125 Catania, Italy; giuseppe.floresta@unict.it (G.F.); chiara.zagni@unict.it (C.Z.); davide.gentile@unict.it (D.G.); vincenzo.patamia@unict.it (V.P.)

**Keywords:** artificial intelligence, machine learning, drug design, COVID-19, structure-based drug design, ligand-based drug design

## Abstract

The recent covid crisis has provided important lessons for academia and industry regarding digital reorganization. Among the fascinating lessons from these times is the huge potential of data analytics and artificial intelligence. The crisis exponentially accelerated the adoption of analytics and artificial intelligence, and this momentum is predicted to continue into the 2020s and beyond. Drug development is a costly and time-consuming business, and only a minority of approved drugs generate returns exceeding the research and development costs. As a result, there is a huge drive to make drug discovery cheaper and faster. With modern algorithms and hardware, it is not too surprising that the new technologies of artificial intelligence and other computational simulation tools can help drug developers. In only two years of covid research, many novel molecules have been designed/identified using artificial intelligence methods with astonishing results in terms of time and effectiveness. This paper reviews the most significant research on artificial intelligence in de novo drug design for COVID-19 pharmaceutical research.

## 1. Introduction

In December 2019, in Wuhan, China, a was patient diagnosed with atypical pneumonia. Just a few months later, on 11 March 2020, the COVID-19 pandemic was announced by the World Health Organization. Today, it is still a considerable concern for humanity [1]. A problematic aspect for clinicians to address is that the disease caused by the virus can cause a broad spectrum of symptoms and disease outcomes. In most cases, the virus results in common influenza-like symptoms (cough, fever, and fatigue) or even remains asymptomatic. However, in 10 to 20% of the patients, inflammation results in more complicated conditions that have resulted in more than 5.5 million deaths to early 2022 [2]. Because of the COVID-19 pandemic, the research on developing new treatments/therapies and vaccines against the virus, including drug repurposing and de novo design, gained remarkable significance and implications at the global level, and time plays a fundamental role in this field [3,4]. Drug development is long and expensive process; it is estimated that during the years 2000 to 2015, the average cost of developing a newly approved drug was over USD 2.5 billion per single approved molecule. Commonly, it takes up to 15 years from the design of a single drug to reach the market, and less than 15% of selected compounds that are tested on humans, are later proved as safe and effective and can be finally used [5,6]. It sounds reasonable that humanity can’t wait 15 years for the development of a new molecule for COVID-19 treatment and in this case, the computational chemistry applied in drug design is helping accelerate research and providing stunning results.

AI refers to machines, mainly computers, working like humans. In AI, machines execute tasks such as speech recognition, solving problems, and learning. Machines can work and act like humans if they have enough instruction and knowledge. AI systems can be divided into four groups based on machines’ ability to use past experiences to predict future decisions: (1) Machine learning refers to AI where machines are not explicitly programmed to perform tasks, but they learn and improve from training automatically. Deep learning is a subdivision of machine learning based on artificial neural networks for predictive analysis. (2) Natural language processing (NLP) is the interaction between computers and human language. (3) Automation and robotics aim to allow monotonous and repetitive tasks to be performed by machines. (4) Machine vision—machines can capture visual information and then analyze it [7]. AI has been used in applications to solve specific problems throughout industry and academia. Like electricity or computers, AI is a general-purpose technology that has a multitude of applications. It has enhanced language translation, image recognition, credit scoring, e-commerce, and many other aspects of our lives.

In the field of computer-aided drug discovery (CADD), the scientific community worldwide is regularly developing new technologies and algorithms to obtain hit compounds in a short time and reduce the overall cost [8]. Nowadays, the introduction of artificial intelligence (AI) and all the related techniques such as deep learning (DL), machine learning (ML), and other classical computational chemistry tools to drug discovery has had a significant effect on the success rate and velocity of novel pharmaceutical identification [9].

AI and classical CADD tools can be used alone or combined to produce new approaches that integrate a broad range of algorithms with enhanced prediction capabilities. CADD is classically classified into two methods: structure-based drug design and ligand-based drug design. Both are faces of the same coin and massively rely on force fields, scoring functions, and algorithms to evaluate and rank the studied molecules’ energy contribution in the targeted macromolecular biological system. While computer-aided structure-based drug design (e.g., docking) depends on the actual 3D structure of the targeted binding site of the targeted receptor protein to understand the stabilizing interactions at the molecular level between the studied ligand/receptor system, the ligand-based drug design (e.g., 3D-QSAR modeling) approach relies on the recognition of a database of already-known ligands interacting with the target receptor. Both structure- and ligand-based technologies have several success stories and play key roles in the drug modern drug discovery process [10,11,12,13,14]. In this context, several of these approaches have recently been employed to research novel drug candidates against COVID-19. The absence of complete protection from vaccination and powerful drugs for the treatment of the infection, the mutability of the virus, and the mortality rate impel the fast discovery of novel molecules active against COVID-19, and CADD is believed to be a valuable tool to achieve this goal [15]. Moreover, AI and ML have been broadly exercised from the beginning of the pandemic in discovering new treatments, vaccines, and drug repurposing, and helping clinicians in pharmaceutical-related big-data analysis and explanation for a better understanding of the outcome of the disease. Ongoing evidence shows that AI is being exploited to find potential novel molecules, repurpose drugs, find novel drug targets, and design novel and more effective vaccines. This review describes the current state of the art and the stunning results of AI and ML in the last two years of research in the field of de novo drug design since the COVID-19 pandemic started. Despite occasional difficulty in classification due to the multidimensional structure- and ligand-based approaches used, we divided the review into three main sections reflecting the application of the AI in each paper (i.e., if most AI used is structure- or ligand-based for the multidimensional approaches), a section regarding de novo vaccine design is also presented.

## 2. Structure-Based Artificial Intelligence Methods for Small Molecules

The SARS-CoV-2 spike protein (S protein) is the leading mediator of viral entry into cells, and thereby infection, by binding the human angiotensin-II converting enzyme (ACE2) and therefore represents an attractive target for drug therapies [16]. Recently, Srinivasan et al. developed a surrogate multi-task neural network (MTNN) model that replaces docking simulation in the finding of new molecules targeting the spike protein [17]. Monte Carlo algorithms and recurrent neural networks (RNN) were used to explore the chemical space of millions of potential molecules using SMILES input. In this way, they discovered 97,973 new molecules, not included in the existing starting databases. The molecules were docked using Vina for their ability to bind the pocket region of the SARS-CoV-2 S-protein/ACE2 complex (SARS-CoV-2 S-protein (NCBI Reference Sequence YP_009724390.1)/ACE2 receptor (PDB ID: 2AJF) [18]. The docking calculation search space was chosen as 1.2 nm × 1.2 nm × 1.2 nm and includes the binding pocket located at the S-protein/ACE2 interface [19].

Several molecules with good scores were selected and compared to FDA-approved drugs and the BindingDB dataset. Despite identifying compounds with great binding affinity, MTNN performance had to be improved using active learning and extending the training data to have a correlation between the SMILES string and Vina scores considering the larger domain of SMILES space. This method increased the number of molecules selected to over 300,000. Of these, 200 promising molecules were chosen using Vina scores as selection criteria.

This method accelerated the exploration of the vast chemical space represented by SMILES strings to evaluate structural features and discover structural similarities between top-performing candidates.

Many of the reported computational studies have selected molecules with high affinity to the spike protein. However, these studies are limited to the receptor ACE2/protein spike interface [19]. This represents a limitation since it dramatically reduces the possibility of identifying potential allosteric inhibitors of ACE2-spike complex formation, leading to identification of a limited number of potentially active compounds.

To overcome this, a new machine learning approach, namely SSnet, was used to identify new potential drugs by screening a library of approved drugs from the DrugBank and ZINC databases [20] targeting two different conformations (open and closed) of the ACE2 receptor as well as ACE2 in complex with the S1 domain of the S protein, that is the protein responsible for binding with human cells [21]. After cross-validation of the hits using the Autodock Vina scoring function [22], the SSnet approach was extended to a library of 750,000 molecules in BindingDB to gain additional information regarding de novo drug design.

After that, to accelerate the identification of high-affinity scaffolds to test for their in vitro activity, a web interface, where molecules are grouped according to their similarity on a 2D map and colored based on binding affinity to the protein, was developed. This system allows selecting a certain point of the interface to explore the effect of singular scaffolds and functional groups on the binding score or affinity. Moreover, this approach can be applied to other therapeutical targets besides COVID-19.

SARS-CoV-2 3CL^pro^ main protease (Picornain 3C-like protease, also referred to as M^pro^ for the main protease) is a homodimeric cysteine protease representing an attractive target for trans-variant activity since no mutations have yet been observed in this protein.

High-throughput virtual screening (HTVS) coupled with ML experiments have been performed to obtain potential virtual inhibitors against the targeted protein rather than trusting commercial “corona-focused libraries”. The system was associated with an ML classification experiment where each compound is indexed into the chemical space of M^pro^ inhibitors, viral protease inhibitors, or a new chemical space. This approach has the advantage of taking into consideration potential drugs that would otherwise have been omitted and gaining information into the possible mechanism of action of the selected compounds [23]. Initially, the ZINC 15 library [24] was employed, and over 9 million compounds with a molecular weight below 200 g/mol were selected. The target SARS-CoV-2 M^pro^ crystal structure downloaded from the database has the PDB ID 6Y7M [25]. HTVS docking was performed using CmDock docking calculations considering the QuickProp, QPlogS descriptor that indicates possible soluble compounds, and 200 hits were selected. A set of M^pro^ inhibitors and viral cysteine protease inhibitors collected in the ChEMBL database with experimental IC_50_ < 100 μM were selected, and a set of 208 chemical descriptors were calculated to organize the compounds from the perspective of their representative chemical space [26].

Using this HTVS method, a set of top-scoring compounds that could inhibit SARS-CoV-2 main protease were identified for further compound prioritization in biological evaluation experiments (Table 1).

A different research group screened a library of molecules for their potential ability to inhibit SARS-CoV-2’s main protease (M^pro^) and the receptor-binding domain (RBD) of the spike protein by using the molecular docking software AutoDock Vina [27]. M^pro^ is a peculiar cysteine protease of the coronavirus family and has a crucial role in mediating viral replication and transcription. The absence of a homologous human protease makes this protein an important target against COVID-19 [28].

The studies included 7675 molecules from the African Natural Product Database (AfroDB) and North African Natural Product Database (NANPDB), 43 FDA-approved antivirals, and 940 compounds derived from a machine learning study on viral M^pro^ [29]. AfroDB and NANPDB 470 were filtered using an ADMET predictor in order to include in the study only the compounds with low toxicity and molecular weight between 250 and 350 g/mol.

**Figure 1:**
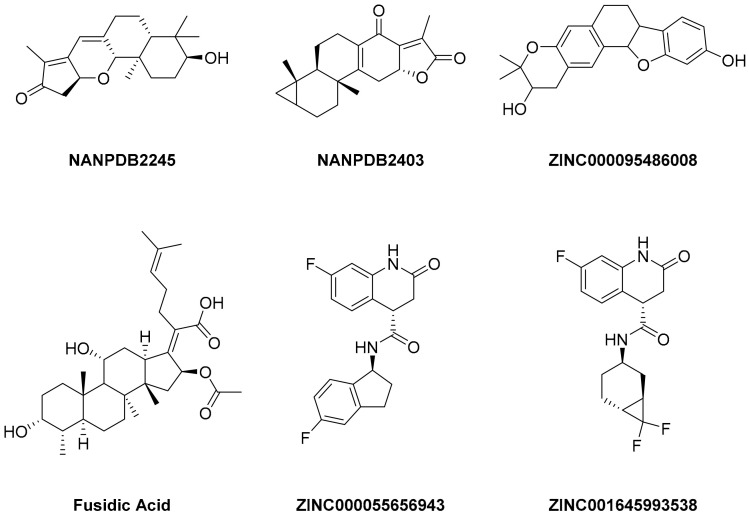
Ligand ID and structures of selected hit compounds.

A library of 2430 compounds was selected and docked for the M^pro^-ligand complex and RBD-ligand complex using Autodock Vina. A total of 36 compounds with binding affinities ≤−7.5 (kcal/mol) against both RBD and M^pro^ were selected and characterized for their binding affinity. After that, a predictor of biological activity using a Bayesian-based approach was accomplished and led to identifying 6 novel potential bioactive molecules. The leads selected NANPDB2245, NANPDB2403, fusidic acid, ZINC000095486008, ZINC0000556656943, and ZINC001645993538 (Figure 1) were subjected to molecular mechanics simulations involving molecular mechanics Poisson–Boltzmann surface area (MM/PBSA) calculations that showed stable protein–ligand complexes with all the compounds with free binding energies <−3.58 kcal/mol with each receptor. The compounds identified showed good pharmacological profiles with little toxicity. However, in vitro studies are still needed to corroborate the findings.

Recently, Born et al., devised a new method for discovering and synthesizing drugs against SARS-CoV-2 [30]. This procedure merges biology and chemistry for target-driven molecular design associated with an automatic synthesis plan generator and can be virtually applied to any protein target. The approach uses deep generative models that implement a conditional molecule generator to propose drug candidates by exploring the latent space of proteins and small molecules. The promise of this approach is the possibility to generalize to unseen targets.

In this way, a conditional molecular generator can produce novel structures expressly designed to target a protein of interest [31]. In this work, 41 SARS-CoV-2-related protein targets, as labeled in UniProt, have been retrieved, among which M^pro^ and spike are the most targeted ones.

The first step of the procedure consists of encoding the selected protein sequence in a continuous and compressed latent space. The latent representation of the profile is decoded through the molecular decoder of trained variational autoencoders (VAE), generating valid molecules as drug candidates. Prediction studies of the toxicity were also performed by employing the Tox21 database [32], and the molecules were screened against 12 toxicity assays of nuclear receptor and stress response pathways. Finally, they were divided into toxic and non-toxic. The selected compounds’ binding affinity was predicted using a multimodal deep learning model that classified compound–protein interaction samples as binding or non-binding.

To assess the feasibility of synthesizing the generated compounds, the retrosynthetic pathways of a subset of candidates for each target were assessed using the interface of IBM RXN (https://rxn.res.ibm.com/, accessed on 10 February 2022) [21]. For half of the generated molecules, the synthetic route could be accomplished in one or two steps starting from commercially available materials indicating that they are attractive drug candidates.

The founders of COVID Moonshot, a non-profit, open-science consortium of scientists from around the world dedicated to discovering globally affordable and easily manufactured antiviral drugs against COVID-19, demonstrated the utility of a de novo design using machine learning with synthesis route prediction [33]. The purpose of the study was to generate potential new drugs targeting the main protease (M^pro^) of the novel coronavirus. In fact, while classic approaches tend to modify existing compounds, exploring limited chemical space, this machine learning method searches in a larger chemical space. The inconvenience of this approach is the expense of synthetic accessibility, but this can be overcome using machine learning to predict the synthetic route.

The learning-to-rank machine learning model [34] here reported consists of a classifier that predicts the activity of a molecule compared to another compound by considering the difference in pharmacophore fingerprint. The output is plotted in a curve reporting the relative activities of the considered compounds. The ligands’ ranking is better than a model that directly learns IC_50_. The model used for training was the FastAI Tabular model (J. Howardet et al., https://github.com/fastai/fastai, accessed on 10 February 2022) with the initial input features composed by Morgan, Atom Pair, and Topological Torsion fingerprints implemented in RDkit (RDKit: Open-Source Cheminformatics Software, https://www.rdkit.org, accessed on 10 February 2022).

After a selection of reasonable chemical perturbations, a fragmentation of synthetically accessible bonds, including amides and aromatic C–C and C–N, were performed, generating 8.8 million molecules that were compared to the most potent molecule in the dataset. The manifold platform was used to predict the synthetic route prediction of the identified compounds (https://postera.ai/manifold, accessed on 10 February 2022). Finally, the best five predicted molecules with no more than 4-step synthesis prediction were synthesized and evaluated for their ability to inhibit M^pro^ by fluorescence assay and are reported in Table 2, together with the most potent molecules obtained from the training set. Compound **16** that showed the best IC_50_ was also tested against OC43 coronavirus in a live-virus assay showing low cytotoxicity and discrete activity toward the virus (EC_50_ = 13 µM).

The Oak Ridge National Laboratory Summit supercomputer was used for in silico drug discovery using enhanced sampling molecular dynamics (MD) and assembly docking using the popular docking program Autodock Vina [35]. The Summit supercomputer is currently the fastest in the United States, hosted at the Oak Ridge Leadership Computing Facility (OLCF). Summit is an IBM AC922 system consisting of 4608 large nodes, each with six NVIDIA Volta V100 GPUs per node. The temperature replica exchange molecular dynamics (T-REMD) routine [36,37], which was chosen here for the MD calculations (see below), uses the interconnect not only to allow for parallelization of a single simulated molecule but also to communicate between separate replicas of the system, each carried out at a different temperature, and performs exchanges between replicas to accelerate the conformational sampling of the structures [38].

The 24 systems analyzed comprise nine protein domains. Two of these, RBD of protein S (spike) and the *N*-terminal region of protein N (nucleocapsid), are structural domains that are bound within the virion. Protein N is used for packaging the viral genome and is essential for virion assembly [39]. The remaining seven domains come from non-structural proteins (NSPs) 3, 5, 9, 10, 15, and 16, which form the replication complex and are involved in many key tasks that create new viral particles (Table 3) [40].

Two different docking databases were used—a potential ligand database merging the contents of SWEETLEADS [41] and the NCI-diversity database, yielding 13,757 compounds, and the Enamine database using an accelerated version of Autodock (Autodock-GPU).

The authors used an experimental screening database of 2900 chemicals tested by the National Institutes of Health, National Center for Advancing Translational Sciences (NCATS) and listed at https://opendata.ncats.nih.gov/covid19/databrowser, accessed on 10 February 2022), to compare with positives identified experimentally by NCATS.

Interestingly, all four experimentally tested compounds (i.e., 100% of the tested compounds in the top 10 lists) were strongly active.

The ensemble docking performed affects database reuse limited to approximately 10,000 compounds. Many of these compounds are expected to be quite promiscuous in binding to targets. Two of the compounds identified in the richest 1% of the preliminary protein S screening have been reported in two registered clinical trials (quercetin and hypericin).

Pirolli D. et al., with the support of machine learning approaches, reported a structure-based virtual screening as an effective strategy to discover inhibitors of protein–protein interactions (PPIs) between SARS-CoV-2 RBD and human ACE2 using the ZINC database [42].

Using different ligand- and structure-based approaches, a customized virtual screening (VS) strategy was set up. The first step of the VS strategy was the selection of a library focused on small molecule PPIs from a dataset of 2 million compounds, using a ligand-based approach capable of recognizing chemical characteristics and scaffolds common to known modulators. For this purpose, a convolutional neural network (cNN) was trained to obtain a QSAR model capable of identifying potential PPI modulators within a virtual library of unknown molecules. The molecules classified by the cNN-based QSAR as potential PPI modulators were further filtered by the expected toxicological properties to discard compounds harmful to human health. The resulting virtual library was hooked to ACE2 to identify compounds with the best binding affinity for the spike protein interaction surface. The dataset of 30,029 ligands obtained from QSAR modeling and toxicity analysis against the druggable site-4 pocket region was used to screen for effective inhibitors of the protein–protein interactions of the SARS-CoV-2 RBD/ACE2 tip. Then a virtual Glide screening in SP mode was performed, and the next phase was done by XP docking. Based on the docking score, the first 15,015 classified ligands (50%) were selected and reassessed with Prime MM-GBSA to estimate their binding free energy. The compounds were then filtered based on distance constraints by selecting only small molecules within 4.5 Å away from any atom of Tyr83 and Gln24 residues. The remaining 9730 molecules were then grouped based on their diversity, and the resulting 973 virtual hit compounds were further assembled into 66 clusters using interaction fingerprints.

The results indicated that most of the compounds screened share a high similarity (0.6–0.7) with the training set.

Four compounds were selected as potential ACE2 surface binders capable of preventing RBD spike recognition and thus infection (Table 4). The presence of an aromatic region facilitates the interaction with ACE2 Phe28 (compounds **2** and **3**) and Tyr83 (compounds **1** and **4**). Furthermore, ACE2 Gln24 and Tyr83 contribute to the stabilization of ligands 1, 2, and 4 within the binding site by forming hydrogen bonds to their hydroxyl groups. Compound **1** is stabilized by the hydrogen bonds formed by the catechol with the oxygen atom Gln24 and the cycloheptyl fraction with Tyr83. Similarly, compound **2** is hydrogen-bonded to Tyr83 and Gln24 oxygen atoms by its hydroxyl and carbonyl oxygen atoms.

## 3. Ligand-Based Artificial Intelligence Methods for Small Molecules

**Figure 2:**
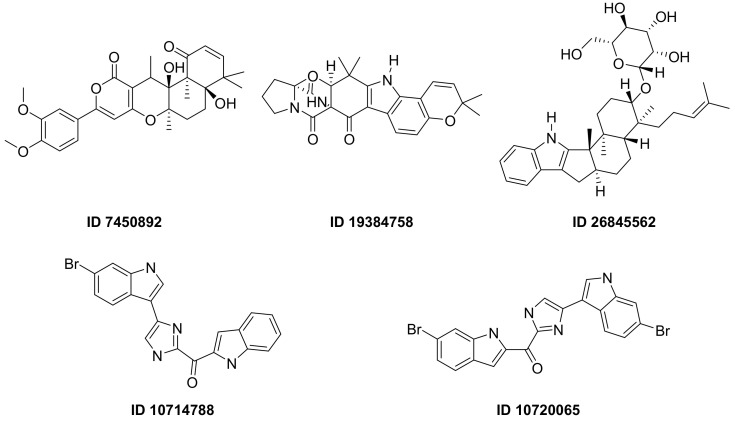
MNPs have been proposed as the most promising marine drug leads as inhibitors of SARS-CoV-2 M^pro^.Combining a generative recurrent neural network model with transfer learning methods and active learning algorithms, R. Yassine et al. designed a novel set of small molecules capable of effectively inhibiting the 3CL protease in human cells [44]. The novelty of this work is the use of active learning methods with generative recurrent neural networks (RNNs) containing long-term memory cells (LSTM). The active learning method facilitates the selection process by focusing on the areas of the chemical space that have the best chance of success, considering structural novelties. The authors built a database consisting of multiple datasets such as FDA-approved drugs (from the ZINC database), natural products (from SuperNatural), and a manually developed database representing drug-like bioactive molecules. In the first phase of this study, by applying the RNN deep learning methodology, the LSTM-based RNN model was created to generate reliable and high-quality SMILES to design new drugs. Subsequently, molecules structurally similar to drugs with known activity against the specific SARS-CoV-2 target were generated. In this way, they were able to find a model capable of discovering new drugs using fragment-based drug discovery (FBDD) to create a library containing a series of SMILES inspired by the well-known compounds. The model generated 25,000 small molecules from the learned chemical space as described above. After removing duplicates and identical molecules from the database used for training, the remaining dataset consisted of 22,173 molecules. These molecules were then subjected to other filters such as physicochemical properties, drug similarity, and synthetic accessibility, resulting in a set of 6962 molecules. The generated molecules were then screened for affinity to the 3CL protease. After the virtual screening, a total of 41 molecules were obtained, with a virtual screening score of less than −7.0 kcal/mol. Among these, four molecules resulted in a binding affinity score lower than −18 kcal/mol (Figure 3).

F. Pereira et al., succeeded in predicting five new inhibitors against SARS-CoV-2 M^pro^ using a CADD method based on a quantitative structure-activity relationship (QSAR) classification model that was built from 5276 organic molecules extracted from the ChEMBL database. Virtual screening was then performed using 11,162 marine natural products (MNPs) retrieved from the Reaxys^®^ database. From the QSAR approach, 494 MNPs were selected and subsequently subjected to molecular docking against the M^pro^. Among the evaluated compounds, five MNPs have been proposed as the most promising marine drugs as inhibitors of SARS-CoV-2 M^pro^, among them a benzo[f]pyrano [4,3-b]chromene (Reaxys ID 7450892), notoamide I (Reaxys ID 19384758), hemindole SB beta-mannoside (Reaxys ID 26845562), and two derivatives of bromoindole (Reaxys IDs 10,714,788 and 10720065) Figure 2 [43].

**Figure 4:**
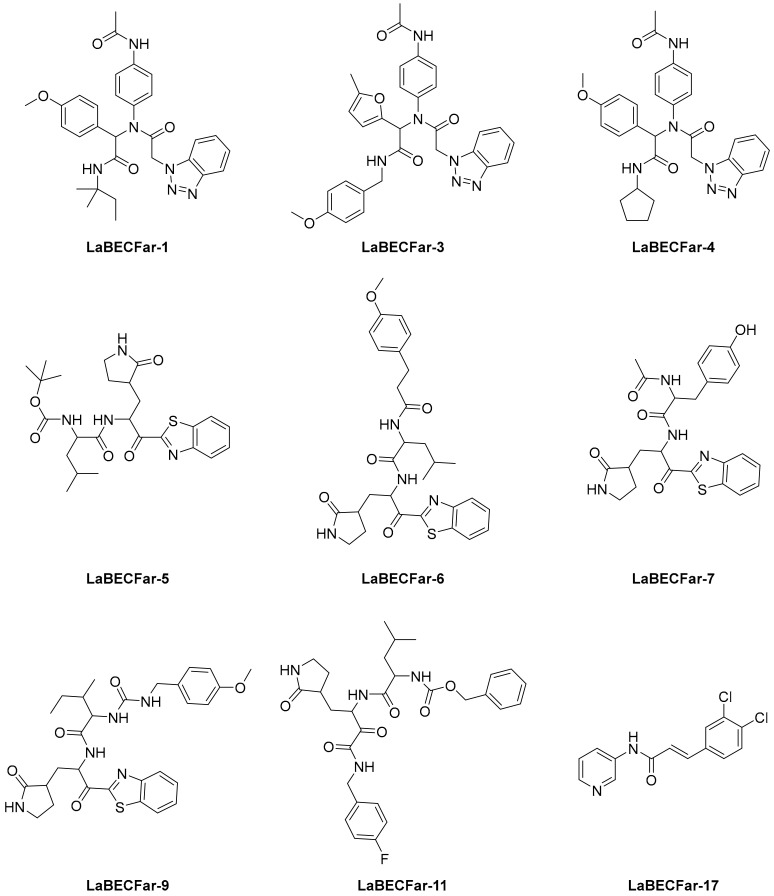
The nine compounds reported by F. P. Silva-Jr et al., that showed similar binding positions to the experimentally validated inhibitors in X-ray crystal complexes with M^pro^.

The reported model developed by F. P. Silva-Jr et al., also outperformed the Chemprop model available for free on an external test set of fragments shielded against SARS-CoV-2 M^pro^ [45]. The method is divided into three main phases—formation and validation of the generative model based on general chemistry, development of the model for M^pro^ chemical space of SARS-CoV-inhibitors, and formation of a classifier for the prediction of bioactivity using transfer learning. Using the improved classifier to predict the bioactivity of the 70,000 valid SMILES, the authors classified 1697 active molecules, and the Uniform manifold approximation and projection (UMAP) plot showed good to optimal overlap between the predicted results and the actual value of inhibition for M^pro^ inhibitors in the studied chemical space. Among the resulting molecules, 20 compounds were classified as high-confidence hits, with probabilities ranging from 0.99 to 1.0. These molecules found to be potential inhibitors were then subjected to a docking simulation using the crystalline structure of SARS-CoV-2 M^pro^ (PDB: 6W79). Nine compounds showed binding poses similar to experimentally validated inhibitors in X-ray crystal complexes with M^pro^. These molecules include three benzotriazoles and four benzothiazolylketones, a peptidomimetic, and an *N*-(2-pyridyl) acetamide derivative (Figure 4).

**Figure 5:**
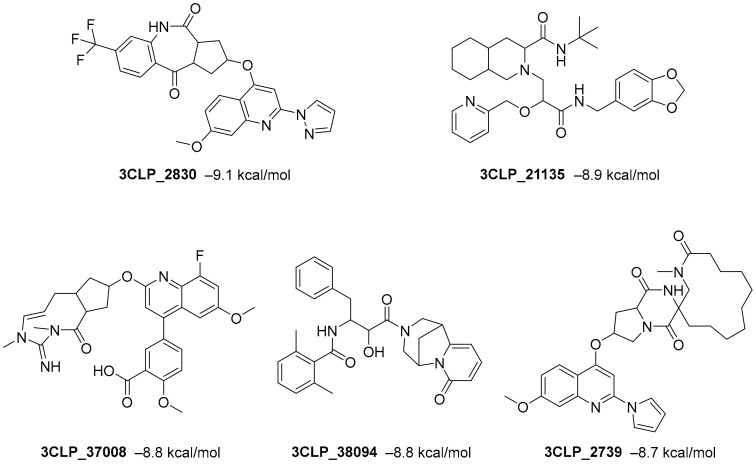
NCEs with the highest virtual screening score and a remarkable similarity to existing protease inhibitors.

Roy et al. recently identified new chemical entities (NCEs) starting from a dataset of approximately 1.6 million drug-like small molecules from the ChEMBL database, which were collected for pre-training of a generative model [46]. The set of molecules obtained after applying the physicochemical property filters was screened using RDKit by applying the following four filters: Pan Assay Interference Compounds, the BRENK filter, the NIH filter, and the ZINC filter. These filters use rules to avoid toxic compounds and synthetically impractical molecules. Potential NCEs for synthesis and testing against SARS-CoV-2 were finally subjected to docking simulation and selected using a virtual screening score cutoff of −8.5 kcal/mol. The results showed that 5 of the top 15 compounds have a high virtual screening score and a remarkable similarity to existing protease inhibitors, notably one of these NCEs possessed a higher virtual screening score of −9.1 kcal/mol (Figure 5).

The unique approach to data curation coupled with random forest (RF) analysis by K. Cooper et al., produced a specific target and validated predictive fingerprints (PFFs) that have a high predictability value on multiple targets such as plasma kallikrein, HIV protease, NSP5, NSP12, AT-1, and the JAK family. This broad applicability to different biologically relevant targets (protease, RNA polymerase, G protein-coupled receptor (GPCR), tyrosine kinase, and a phenotypic assay) as well as to different chemotypes within a target is an important strength and differentiator of the applied methodology. The capability of this methodology allows for each target to create a binary decision tree for inactive or active compounds or a ternary decision tree for weakly active, moderately active, and highly active compounds also suggests that the models could be used for virtual screening of libraries of target-specific compounds to select the most active compounds for synthesis and/or clinical testing. Regarding the SARS-CoV-2 target, about 5600 FDA-approved drugs were examined in this study. Molecules that showed more than 75% inhibition were considered active, and among the wide range of FDA-approved drugs, 267 were identified as active. Leveraging the bioactivity data of ChEMBL and a subset of the data, it was trained on physicochemical characteristics from the set of 110 chemical properties, and a set of 868 compounds was then used to establish a binary classification model capable of predicting whether a molecule was active or inactive in the test; overall the model was able to identify the bioactivity with an accuracy of 65% [47].

E. Glaab et al., reported a combined virtual screening study, molecular dynamics (MD) simulation, machine learning, and in vitro experimental validation analysis, which led to the identification of small molecule inhibitors of 3CL^pro^ with micromolar activity and to a pharmacophore model. The methodology consisted of a filtering system involving screening multiple receptors and ligands in combination with a final MD simulation to confirm the binding stability of the selected compounds. The structural screening was then integrated with machine learning-based screening for compound selection using a molecular-descriptive dataset derived from known ligands and non-ligands for 3CL^pro^. The best compounds selected using these in silico screening methods were then experimentally evaluated to determine the subset of stable ligands and their 3CL^pro^ inhibitory activity using the in vitro Forster-type resonance energy transfer (FRET) assay. From the various screening analyses conducted, 95 molecules were identified and tested. Seven of these compounds were confirmed active, and one of them showed the lowest IC_50_ value of 31 µM [48].

The deep learning (DL) model can generate 1D or 2D sequences ligands structures. However, rational drug design requires 3D ligand structures that target the crystalline structure of proteins. To solve this problem, Q. Bai and coworkers developed MolAICal software, which allows to generate 3D drugs in the 3D pocket of protein targets by combining the merits of the deep learning model and classical algorithm. The software essentially consists of two modules. In the first one, FDA-approved drug fragments are used to train the Wasserstein-based deep learning model generative adversarial networks (WGANs). The generated fragments of the deep learning model are further used to grow the 3D ligands in the protein pocket. In the second module, drug-like molecules from the ZINC database are used to train the WGAN-based deep learning model. Then the affinities between the generated molecules and the proteins are evaluated through molecular docking experiments with AutodockVina. The membrane protein glucagon receptor (GCGR) and the non-membrane target SARS-CoV-2 M^pro^ were chosen to analyze the drug design capabilities of MolAICal. In this way, the software can generate various ligands that have an ever-higher 3D structural similarity with the ligand crystallized in the active site of GCGR or SARS-CoV-2 M^pro^ [49].

Mekni N. et al., developed a machine learning approach using support vector machine (SVM) classification, to share new knowledge for designing novel M^pro^ inhibitors from a dataset of two million commercially available compounds. The model was able to classify two hundred new chemotypes as potentially active against the viral protease [50].

Feature selection was made by implementing a python3 script using the Sklearn libraries. The script is available in the GitHub repository (https://github.com/NedraMekni/COVID-19, accessed on 10 February 2022).

The selection of the characteristics was based on the training set, with the aim of identifying the crucial molecular descriptors able to explain the possible correlation between the activity of the M^pro^ inhibitors and their chemical structures, implemented the elimination of the recursive characteristics of the random forest (RF-RFE) in order to select relevant molecular descriptors [51], leading to the automatic optimization of the number of features to be selected and to the definition of the optimal number of decision trees to build the forest. Compounds labeled as active by SVM were subsequently evaluated through consensus docking studies on two PDB structures, and their binding modes were compared with known protease inhibitors. Of the 25 facilities analyzed, only five (5RF6, 5RGW, 6WCO, 5R82, and 6W79) met the criteria. On these five PDBs, factor B (mean of the PDB B-value) was checked to assess the quality of the protein structure.

The five best compounds selected by consensus were then subjected to molecular dynamics to investigate the stability of the binding interactions.

MD simulations at 200 ns were performed on the two best performing PDBs (6WCO and 5RGW) to verify the stability of the interactions recovered within the crystal structure.

The five best compounds selected by consensus were then subjected to molecular dynamics to investigate the stability of the binding interactions. It should be noted that the compounds selected by SVM showed all the essential interactions reported in the literature.

Joel Kowalewski and Anandasankar Ray, collected test data from 65 known human target proteins that could interact with SARS-CoV-2 proteins, including the ACE2 receptor [52]. Next, they trained machine learning models to predict the inhibitory activity and used them to examine FDA-registered chemicals and approved drugs (~100,000) and ~14 million purchasable chemicals. The results were then filtered for mammalian toxicity and vapor pressure. Potential volatile candidates were proposed as drugs for inhalation therapies as the nasal cavity and airways are the first bottlenecks for infection. They also identified some candidates that may act on multiple targets.

S. Ekins et al., implemented several machine learning methods to develop predictive models from recent in vitro SARS-CoV-2 inhibition data and used them to prioritize additional FDA-approved compounds for in vitro testing selected from our in-house library of compounds [53]. From the compounds predicted using a Bayesian machine learning model, lumefantrine, an antimalarial, was selected for testing and showed limited antiviral activity in cells, whereas it was shown to bind (*K*_d_ 259 nM) to the spike protein using microscale thermophoresis. Several other selected compounds were tested in vitro by other research groups and found to be quite active. This combined machine learning approach and in vitro testing can be expanded to virtually screen available molecules with predicted activity against SARS-CoV-2 WIV04 and other circulating variants.

## 4. Artificial Intelligence Methods Vaccine Design

An in silico deep learning approach was proposed to predict and design a multiepitope vaccine (DeepVacPred), in combination with in silico immunoinformatics and deep neural network strategies. The DeepVacPred computing system directly predicted 26 potential vaccine subunits from the SARS-CoV-2 tip protein sequence [54].

The DNN architecture to block 26 fragments in the SARS-CoV-2 spike protein as candidates for the vaccine subunit was the first step proposed by the authors. Subsequently, linear B cell, CTL, and HTL epitopes were used to select and construct the final vaccine.

All overlapping protein fragments with a length of 30 aa were generated by the spike protein sequence 1273 aa SARS-CoV-2. DeepVacPred first tested these protein sequences and predicted 132 potential vaccine subunits. Following this prediction, DeepVacPred provided 26 potential vaccine subunits for further evaluation and construction. These subunits were those most likely to contain B cell epitopes and multiple T cell epitopes and have high antigenicity and low allergenicity.

In silico methods were used to study linear B cell epitopes, cytotoxic T cell epitopes (CTL), helper T cell epitopes (HTL) in the 26 candidate subunits.

The B cell epitopes, predicted on the 26 vaccine subunits, are parts of antigens that bind to immunoglobulin or antibody, capable of activating B cells to provide the immune response [55]. Linear B cell epitopes were predicted from four online servers, including BepiPred [56], SVMtrip [57], ABCPred [58], and BCPreds [59]. First, they used BepiPred for the main forecast and the other three servers to check the results of the BepiPred forecast. Additionally, the proprietary RaptorX server was used to evaluate the surface accessibility of SARS-CoV-2 to validate that the B cell epitopes in those subunits were well exposed.

CTLs recognize infected cells using class I MHCs to bind to certain CTL 26 epitopes. The NetMHCpan 4.1 server [60] 43 was used to predict potential CTL epitopes. All overlapping 9aa peptide sequences in the 14 vaccine subunits were tested with the 12 most common class I alleles of human leukocyte antigen (HLA), including HLA-A1, HLA-A2, HLA-A3, HLA-A24, HLA-A26, HLA-B7, HLA-B8, HLA-B27, HLA-B39, HLA-B44, HLA-B58, and HLA-B62, to evaluate their binding affinities and predict potential CTL epitopes [61,62].

HTL helps other immune cells’ activity and recognizes infection by using MHC class II to bind with specific HTL epitopes [63]. The NetMHCIIpan 4.0 [64] server was used to predict potential HTL epitopes. All overlapping 15aa peptide sequences in the 14 vaccine subunits were tested with the 13 most common HLA Class II alleles, including HLA-DRB1-0101, HLA-DRB1-0301, HLA-DRB1-0401, HLA-DRB1-0701, HLA-DRB1 -0801, HLA-DRB1-0901, HLA-DRB1-1001, HLA-DRB1-1101, HLA-DRB1-1201, HLA-DRB1-1301, HLA-DRB1-1401, HLA-DRB1-1501, and HLA-DRB1-1601, to evaluate their binding affinities and predict potential HTL epitopes [64]. The total HLA score was calculated for each vaccine subunit.

The 3D structure of the designed vaccine was then predicted, refined, and validated by other in silico tools. The GalaxyRefine [65] server was employed to refine the 3D structure model of the final vaccine. Among the five refined models predicted by GalaxyRefine, model 2 was chosen as the final vaccine model based on its quality scores with a reported RMSD of 0.58.

In conclusion, this proposed artificial intelligence (AI)-based vaccine discovery facility accelerated the vaccine design process and built a 694 aa multiepitope vaccine containing 16 B cell epitopes, 82 CTL epitopes, and 89 HTL epitopes, which promise to fight SARS-CoV-2 viral infection with good antigenicity, population coverage, and good physicochemical properties and structures, providing great potential for next stage of COVID-19 vaccine design with actual clinical trials.

AI was used in another study to predict the rationale for designing universal vaccines against SARS-CoV-2, which contain an extensive repertoire of T-cell epitopes capable of providing coverage and protection to the global population. To achieve these goals, the authors profiled the entire SARS-CoV-2 proteome through the 100 most frequent HLA-A, HLA-B, and HLA-DR alleles in the human population, using the presentation of the cell surface antigen infected with host and immunogenicity predictors from the NEC Immune Profiler suite of tools and generated sufficiently complete epitope maps [66].

Antigen (AP) presentation was predicted by a machine learning model that integrates information from several HLA binding predictors (in this case, three distinct HLA binding predictors trained on IC_50_ nm binding affinity data) and 13 different antigen processing predictors. The emitted AP score ranged from 0 to 1 and was used as an input to calculate immune presentation (IP) through the epitope map, penalizing those peptides that have degrees of similarity when compared to the human proteome, and rewarding peptides that are less similar. The resulting IP score represents those presented HLA peptides that can be recognized by circulating T cells.

Epitope maps were created for all viral proteins and an example based on IP scores for proteins containing the candidate CD8 and CD4 epitopes for the 100 most frequent human HLA-A, HLA-B, and HLA-DR alleles.

Epitope hotspots that shared significant homology with proteins in the human proteome were removed to reduce the possibility of inducing off-target autoimmune responses. In addition, the antigen presentation and immunogenic landscape of all non-synonymous mutations in 3400 different virus sequences in the GISAID database with the AP potential of the Wuhan Genbank reference sequence were also analyzed to identify a trend by which SARS-CoV-2 mutations are expected to have a reduced potential to be introduced by host-infected cells and, consequently, be detected by the host’s immune system.

In order to assess whether epitope hotspots are solid enough across sequenced and mutant strains of SARS-CoV-2, the AP-based Monte Carlo epitope hotspot statistical model was used, and 10 virus sequences were analyzed among the 10 most mutated viral sequences across different geographical regions [67]. Most of the hotspots were present in all sequenced viruses; however, the hotspots were eliminated and/or new hotspots emerged in these divergent strains.

This is the first computational approach to generate vaccine designs from large-scale epitope maps of SARS-CoV-2, optimized on diverse T-cell immune responses across the global population.

Finally, an HLA haplotype database of approximately 22,000 individuals was evaluated to develop a “digital twin” simulation to model the effectiveness of different combinations of hotspots in a diverse human population; the approach identified an optimal variety of epitope hotspots that could provide maximum coverage in the global population.

The CD8 epitope maps of these optimized epitope hotspots are based on AP predictions of the peptides presented on the surface of the host-infected cells and visible to the host’s CD8 T cells. Furthermore, these antigen-presented peptides are subject to IP predictions, which infer the epitopes most likely to activate a T cell.

In conclusion, the authors combined antigen presentation at the infected host cell surface and immunogenicity predictions from the NEC Immune Profiler with a robust Monte Carlo and digital twin simulation in order to delineate the entire SARS-CoV-2 proteome and identify a subset of epitope hotspots that could be exploited in rational vaccine design to provide broad coverage across the global population [68].

Kesarwani et al., examined data acquired from proteomic analyses of human cell lines infected with SARS-CoV-2 and COVID-19 patient samples to identify peptides useful for diagnostics and vaccine development. Initially, a large-scale meta-analysis of changes in 358,558 SARS-CoV-2 protein sequences detected in samples from 42 countries was performed. For sequence conservation analysis, a protein data cluster was generated for each SASR-CoV-2 protein. Hence, there were 5 regions and 14 regions for the nucleocapsid and spike proteins, respectively [69].

Two cell lines and four proteomes from naturally infected human patients were used for high-confidence identification of peptides using viral and human protein sequences as references. In total, 361 and 81 peptides of viral origin were identified in cell lines and patient samples, respectively. A few peptides with varying lengths were found from different parts of the same proteins, including 57 component peptides of the spike protein. Of these 57 peptides, three are components of the S1 (14–685), S2 (686–1273), and RBD (319–541) regions of the spike protein, respectively.

Therefore, the authors explored host responses to the virus in both cell lines (colon carcinoma-2 and H1299) and naturally infected COVID-19 patient samples. Many proteins are involved in biological processes related to the immune system, such as regulation of immune responses, leukocyte migration, autophagy, processing, immune system development, antigen presentation, or leukocyte-mediated cytotoxicity.

323 and 143 human peptides were identified in the cell line and patients, respectively. Only five (MDGA1, PIK3C2A, FOXP2, DCAF5, and IVD) were detected in both sample sets. While MDGA1 plays an important role in inhibitory synapse formation [70], PIK3C2A is involved in several intracellular traffic and signaling pathways [71]. FOXP2 is a transcription factor that can regulate hundreds of genes in different tissues, including the brain [72]. DCAF5 is a receptor of the CUL4-DDB1 E3 ubiquitin-protein ligase [73], and IVD is an enzyme essential for the beta-oxidation of mitochondrial fatty acids.

Thirty-three proteins were found and matched entries in the InnateDB database. Most of these proteins are involved in immune-related functions such as protein binding (TAB1, SREBF2, HSP90AA1, RB1, STAT3, DCN, IL1R1, BNT3A2, PIK3R2, CCR6), transferase activity (TREM2, ABL1, S100A12, C4BPB), the protein dimerization (UBE2N, CSF1R) and lipopeptide binding (EPS8, CD36) [74].

Once the proteins related to the immune system process were identified from the patients’ cell line and proteomes, they were used to generate a protein interaction network. The generated protein interaction network, which includes 403 nodes and 671 edges, identified higher-ranking hubs and bottlenecks.

Therefore, multi-step filtering was applied to identify potential diagnostic peptides. Initially, to avoid cross-reactivity, the identified peptides (442) were filtered to exclude peptides from the human and human saliva microbiome (418) and subsequently peptides from a targeted group of pathogenic bacteria and viruses (129). Subsequently, using the results of the RNA-Seq data analysis of the infected cell lines, the expression of the selected peptides was verified to avoid the selection of poor peptides for diagnostic purposes. Then, four antigenic peptides were selected for attachment to known viral T-cell receptor (TCR), class I and II peptide major histocompatibility complex (pMHC), and paratopes sequences identified. They also tested the paratope binding affinity of SARS-CoV-1 T and B cell peptides that had previously been experimentally validated. The resulting antigenic peptides have a high potential for generating antibodies specific to SARS-CoV-2, and the peptides of the paratopes can be used directly to develop a COVID-19 diagnostic assay

The antigenic peptides found in this study have a high potential for generating antibodies specific to SARS-CoV-2, and the paratopes peptides can be used directly to develop a COVID-19 diagnostic assay. In addition, the paratope binding affinity of SARS-CoV-1 T and B cell peptides that had previously been experimentally validated was also tested. The assembled paratopes showed a greater binding affinity for SARS-CoV-2 antigens and proteins than SARS-CoV-1.

In conclusion, the authors in this study explored both the cell line and proteomes of naturally infected COVID-19 patients via in silico methods and identified four SARS-CoV-2 antigens and three antigen-binding peptides that could be used to develop diagnostic assays. The proposed antigenic peptides can be used for antibody generation, and the paratopes sequences can be used directly for COVID-19 diagnostic test and vaccine development.

## 5. Conclusions and Perspective

AI technologies have shown impressive ability in COVID-19 research, from real-time tracking of the virus spread to the development of novel drugs and vaccines faster than ever before. Unfortunately, the high number of positive cases and deaths from COVID-19 infection and the absence of effective treatments and complete cover by vaccination persist in influencing global health, necessitating the discovery of novel molecules for the cure and prevention of the infection. Structural definition of targetable proteins for therapy and prevention of the infection has recently boosted the structure-based virtual identification of small molecules. The discovery of some promising compounds has driven the optimization of those scaffolds through ligand-based drug design.

In this review, we reported and discussed the more cutting-edge technologies in the field of COVID-19 de novo drug design, from ligand-based AI technologies, where only the ligands’ structures are considered to train the models, to structure-based AI technologies, where the protein target is also taken into account in the process of drug design, and to vaccine design aided by AI. ML on its own and other AI-based technologies are of critical importance in responding to problems in COVID-19 research. They are proven to be efficient tools to quickly analyze large amounts of data, estimate drug repurposing against COVID-2019, identify the association of these repurposed drugs, or estimate dosage adjustments and other clinical issues such as early diagnosis, identifying people at risk, and predicting disease evolution [75,76].

The de novo drug design field is also part of the AI-lead research in the COVID-19 field. As reported in this review, several molecules have already been identified from impressively large databases of billions of compounds. Additionally, pairing different approaches considering all the information produced by omics sciences should lead to developing personalized strategies. Due to the mutability of this RNA virus and the emergence of drug resistance problems, it is mandatory to start considering targeting multiple targets which may be more effective and help in overcoming future drug resistance.

Interestingly, although none of the de novo-identified molecules has entered clinical trials, some of the repurposed molecules identified by AI technologies have entered this phase. These are mainly already used and approved antibiotics, anti-inflammatory, antivirals, anticancer, and ACE2, and some other drugs, that are already in clinical trials according to ClinicalTrials.gov (https://clinicaltrials.gov/, accessed on 10 February 2022) [77]. In conclusion, as the pandemic crisis has exponentially accelerated the adoption of analytics and AI, it is not surprising that AI leads the research on prevention, not only of COVID-19-related issues but also many for other diseases in the following decade, speeding up the drug design process and placing AI at the forefront of the battle against public health problems.

## Figures and Tables

**Figure 3:**
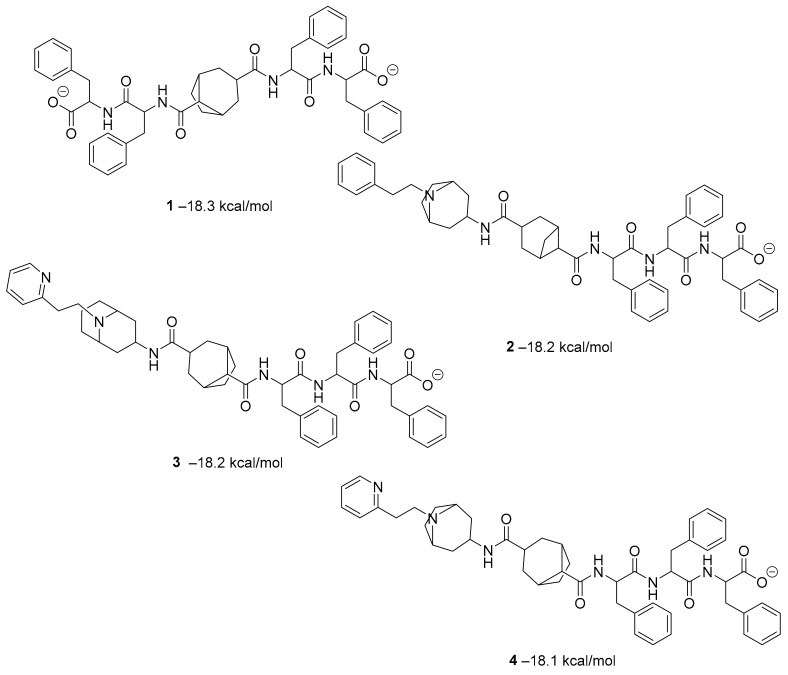
The generated molecules by R. Yassine et al., with the lowest binding affinity scores.

**Table 1:** Selected top-scoring compounds by HTVS on the SARS-CoV-2 main protease.

*n*	Structure	CmDock Docking Score
**1**	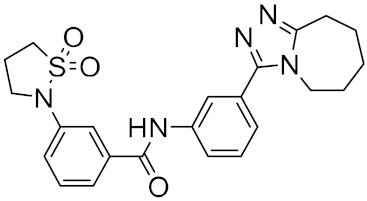	−32.51
**2**	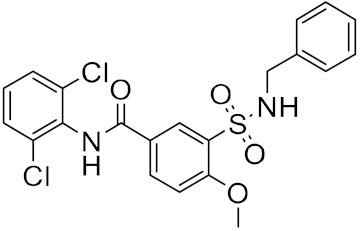	−29.02
**3**	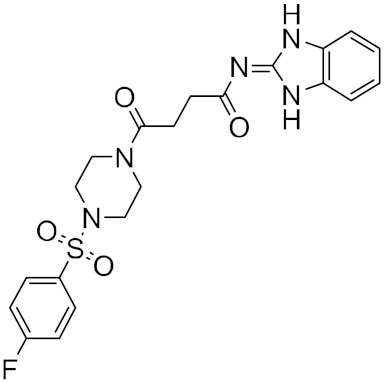	−26.80
**4**	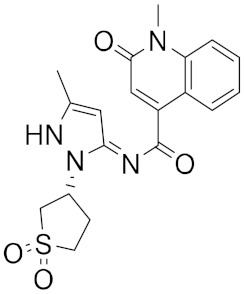	−25.58
**5**	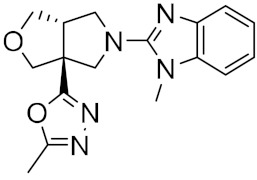	−25.53
**6**	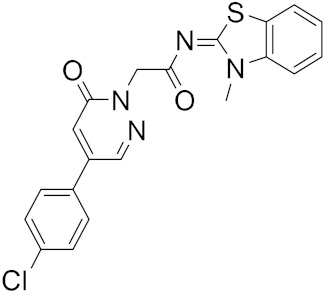	−25.05
**7**	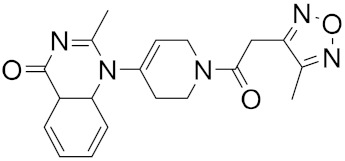	−24.76
**8**	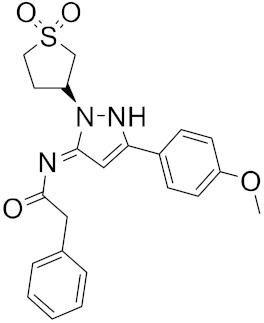	−24.51
**9**	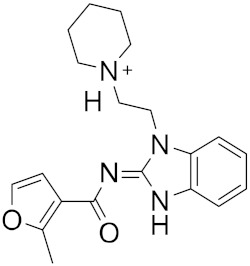	−24.17
**12**	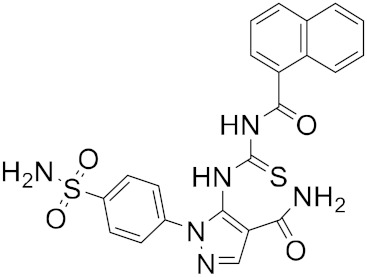	−24.01
**11**	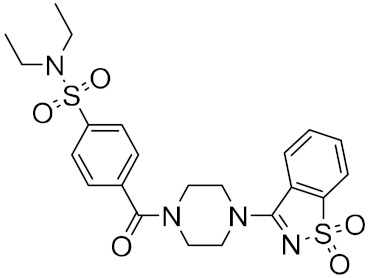	−23.98
**12**	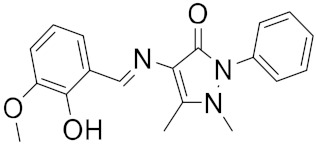	−23.61
**13**	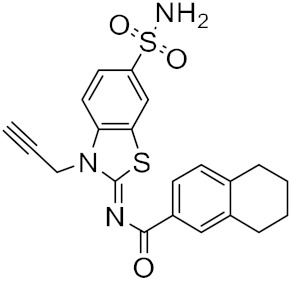	−23.53
**14**	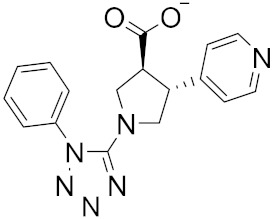	−23.26
**15**	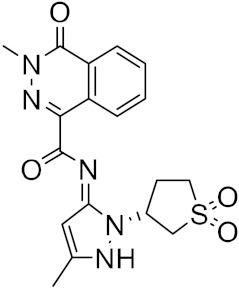	−23.18

**Table 2:** The 5 compounds with predicted routes < 4 steps. The top 3 compounds from the training set, with potency and cytotoxicity measurements.

Top 5 predicted compounds
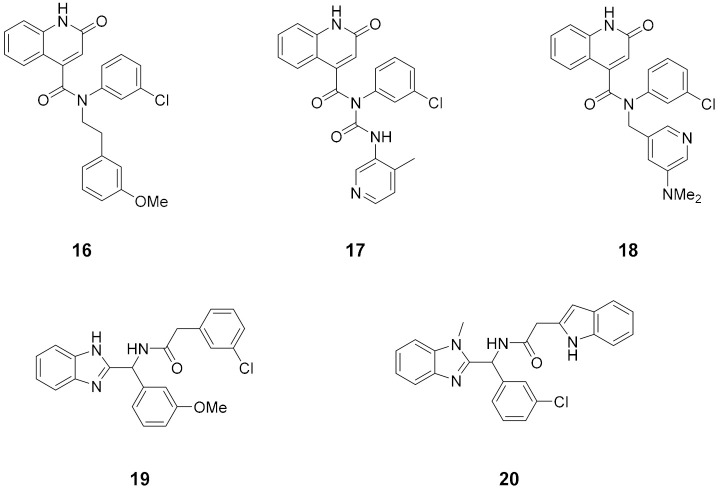
Top 3 training set compounds
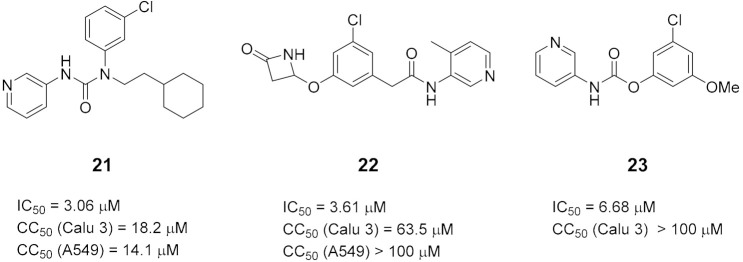

**Table 3:** 24 different model systems.

	Protein/System (PDB Code)	
S (spike) Protein Receptor Binding Domain (RBD)/“Apo” (PDB ID: 6W41) M^Pro^/dimer, CHARMM-GUI default protonation (PDB ID: 6Y2E) M^Pro^ dimer/HIE protonation variant (PDB ID: 6WQF) NSP15 (endoribonuclease)/hexamer (PDB ID: 6VWW) NSP10/monomer (PDB:6W4H) N (nucleocapsid) *N*-terminus phosphoprotein/tetramer (PDB ID: 6M3M) NSP9/monomer (PDB ID: 6W4B) PL^Pro^/monomer “charged” protonation variant (PDB ID: 6W9C)	S Protein RBD/Complexed with ACE2 (PDB ID: 6W41) M^Pro^/dimer, “charged” protonation variant (PDB ID: 6WQF) M^Pro^ monomer/HID41 protonation variant (PDB ID: 6WQF) NSP15 (Endoribonuclease)/monomer (PDB ID: 6VWW) NSP16/monomer (PDB:6W4H) N (nucleocapsid) *N*-terminus phosphoprotein/ tetramer complexed with Zn (PDB ID: 6YVO) NSP9/dimer (PDB ID: 6W4B) PL^Pro^/monomer “neutral” variant (PDB ID: 6WRH)	M^Pro^/monomer, CHARMM-GUI default protonation (PDB ID: 6Y2E) M^Pro^ monomer/HIE41 protonation variant (PDB ID: 6WQF) M^Pro^ dimer/HID41 protonation variant (PDB ID: 6WQF) NSP10:NSP16 Complex (Methyltransferase) (PDB ID: 6W4H) N (nucleocapsid) *N*-terminus phosphoprotein/monomer (PDB ID: 6M3M) N (nucleocapsid) *N*-terminus phosphoprotein/monomer alternate crystal structure (PDB ID: 6YVO) NSP3 ADP ribose phosphatase/asymmetric unit (PDB ID: 6W02) NSP3 ADP ribose phosphatase (PDB ID: 6W02)

**Table 4:** Structures and calculated free binding energies (MM-GBSA score, in kcal/mol) of four top-ranking compounds.

Name	Structure	IUPAC Name	MM-GBSA Score (kcal/mol)
Compound **1**	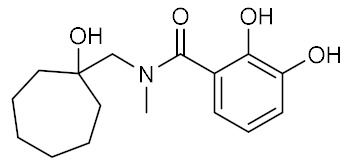	*N*-[(1-hydroxycycloheptyl)methyl]- *N*-methyl-2,3-dihydroxybenzamide	−42.21
Compound **2**	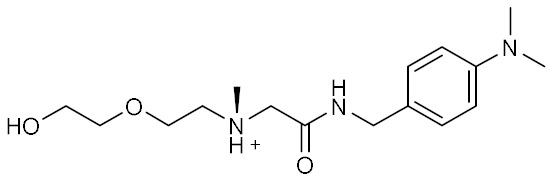	[({[4-(dimethylamino)phenyl]- methyl}carbamoyl)- methyl][2-(2-hydroxyethoxy)ethyl]- methylazanium	−42.07
Compound **3**	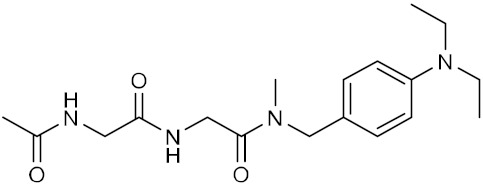	*N*-[({[4-(diethylamino)phenyl]- methyl}(methyl)carbamoyl)- methyl]-2-acetamidoacetamide	−40.95
Compound **4**	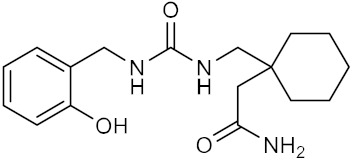	2-{1-[({[(2-hydroxyphenyl)methyl]- carbamoyl}amino)methyl]- cyclohexyl}acetamide	−39.5

## Data Availability

Data sharing not applicable to this article as no data sets were generated or analyzed during the current study.
